# A Prospective, Blinded Study Comparing In-hospital Postoperative Pain Scores Reported by Patients to Nurses Versus Physicians

**DOI:** 10.7759/cureus.6122

**Published:** 2019-11-11

**Authors:** Devon Foster, Glenn Shi, Elizabeth Lesser, Michael G Heckman, Joseph Whalen, Antonio J Forte, Benjamin K Wilke

**Affiliations:** 1 Miscellaneous, Mayo Clinic, Jacksonville, USA; 2 Orthopedics, Mayo Clinic, Jacksonville, USA; 3 Emergency Medicine, Mayo Clinic, Jacksonville, USA; 4 Plastic Surgery, Mayo Clinic Florida Robert D. and Patricia E. Kern Center for the Science of Health Care Delivery, Jacksonville, USA

**Keywords:** pain, numerical rating scale, opioids, pain scales, pain control

## Abstract

Introduction: Referred to as the “fifth vital sign”, pain is unique in that it cannot be obtained accurately by objective measurements. Instead, providers rely on patient-reported scales, such as the numerical rating scale (NRS), to determine a patient’s pain level. Research has shown that patients report different pain scores to nurses and physicians in the clinic setting. It is unknown if this also occurs in the acute postoperative period. We hypothesized that patients report similar pain scores to the nursing staff and physician postoperatively. The primary aim of this study was to examine the degree of agreement between these patient-reported pain scores.

Methods: A prospective study was conducted on 90 postoperative patients. During rounds, the surgeon collected a patient-reported pain score using the 11-point verbal NRS. Following rounds, the nursing staff obtained a pain score using the same scale. The patient was blinded to the study.

Results: The median score reported to both the surgeon and nurses was 3 (range: 0-10), with a median difference of 0 (range: -2.5 to 7). Fifty-four percent of patients reported the same score to both the surgeon and the nurse and 88% of patients reported scores within a 1-point difference. This corresponded to an interclass correlation coefficient of 0.90, indicating very good agreement. The degree of agreement in pain scores reported to surgeons and nurses was consistent according to sex and age.

Conclusion: The results of the study demonstrate a high degree of agreement between the pain scores reported by the patients to both the nursing staff and the surgeon postoperatively, with 88% of the scores at most being 1-point different.

## Introduction

Effective pain control has long been a priority in patient care. Often referred to as the “fifth vital sign”, pain is unique from the more traditional vital signs in that it cannot be easily obtained accurately by objective measurements. Instead, medical providers rely on several patient-reported subjective pain scales, such as the visual analog scale (VAS) or numerical rating scale (NRS) to determine a patient’s pain level [1-4]. However, previous studies have reported that medical providers often disagree with the patient’s stated pain level [5-6].

There is additional concern with relying solely on these subjective measurements for pain as patient care is often directed from these self-reported scores. Previous research has demonstrated that patients report different pain scores to the nursing staff and physician during the same clinic visit [7-8]. It is currently unknown if patients report a consistent pain score to different members of the medical team in the postoperative setting. This becomes important as physicians often make medical decisions and changes to medications based on reports from the nursing staff. If patients report a different pain score to different members of the medical team, it may cloud the clinical picture and adversely affect patient care. 

The primary aim of this study, therefore, was to evaluate the degree of agreement between the patient-reported pain score provided to the surgeon as compared to the score provided to the nursing staff in a blinded fashion. Additionally, the secondary aim was to assess the association between the reported pain score and whether the patient felt that their pain was adequately controlled. We hypothesized that patients would report similar pain scores to the nursing staff and physicians postoperatively.

## Materials and methods

Following the Institutional Review Board (IRB) approval, we conducted a prospective single-blinded study to compare patient-reported pain scores provided to the physician as compared to the nursing staff. The data was collected on 90 postoperative patients during inpatient surgical rounds at the Mayo Clinic in Florida from June to September 2018. 

The baseline characteristics that were collected included age, gender, and pre-operative NRS pain score. The pre-operative pain score was collected in the preoperative holding bay by the perioperative nursing staff on the day of surgery. Postoperative measurements recorded included pain scores reported to the surgeon, pain scores reported to the nurse, the time between physician-assessed and nurse-assessed pain scores, and whether the patients’ pain was adequately controlled (as reported by the patient to the surgeon). The primary outcome measure was the difference between pain scores reported to the surgeon and nurse (i.e., nurse NRS pain score minus surgeon NRS pain score). 

Pain scores were collected on average on postoperative day 1 (range: 1-5 days). During rounds, the physician collected the data by asking the patient to state their current pain level on an 11-point verbal NRS, with 0 equaling “no pain” and 10 equaling “the worst pain”. After the patient had given a rating for their pain level, the physician then asked if the patient considered the pain to be currently controlled (yes or no). The time that the patient responded to the physician was recorded. The nursing staff was not present during the physician collected data.

Once the surgical team finished daily inpatient rounds, a researcher returned to the surgical floor and asked the nursing staff to enter the room and obtain a patient-reported pain score using the same 11-point verbal NRS scale. No member of the surgical team, including the researcher, were in the room or in sight of the patient during the nurse’s collection of data to eliminate bias. The nurse-collected scores were obtained within 60 minutes following the physician-collected data, and were prior to any pain-inciting events, such as physical therapy or movement from the bed to chair.

The patients were blinded to the study until after the data was collected, after which they were informed of the study by the research team and consented to include their data in the analysis. No patients refused to consent. All patients included in the cohort were able to consent themselves and were free of a diagnosis of delirium or dementia. For patients undergoing multiple staged procedures, only the first operative orthopedic procedure and first recorded NRS pain scores for each patient were included in the analysis in order to satisfy the statistical assumption of independent measurements.

The median patient age in the cohort was 66 years (range: 19 - 101 years) and 40 patients (44%) were male. The median pre-operative NRS pain score was 0 (range: 0 - 10), and 51 patients (57.3%) had a value equal to zero. The majority of patients underwent an elective operation such as a hip or knee replacement procedure (65 of 90, 72%). Additional procedures included a hemiarthroplasty or cannulated screws for a femoral neck fracture (8 of 90, 9%), soft tissue mass excisions (4 of 90, 4%), pelvic debridement procedures (3 of 90, 3%), and irrigation and debridement for infection (7 of 90, 8%).

Statistical analysis continuous variables were summarized with the sample median and range, while categorical variables were summarized with number and percentage. Agreement in NRS pain scores as reported to the surgeon and nurses was assessed for the overall series as well as separately according to sex and age (<65 or ≥65 years). To estimate this degree of agreement in pain scores, we first estimated the proportion of patients for whom pain scores reported to the surgeon and nurses were equal or within one point of each other. Subsequently, the agreement between surgeon and nurse NRS pain scores was estimated using the intraclass correlation coefficient (ICC) along with a 95% confidence interval (CI); an ICC equal to 0 indicates no agreement and an ICC equal to 1 indicates perfect agreement.

To evaluate the secondary aim of the study, NRS pain scores reported to the surgeon were compared according to whether the patients’ pain was controlled or not using a Wilcoxon rank-sum test. All statistical tests were two sided. P-values less than 0.05 were considered as statistically significant. Statistical analysis was performed using R Statistical Software (version 102 3.4.2; R Foundation for Statistical Computing, Vienna, Austria).

## Results

**Figure 1:**
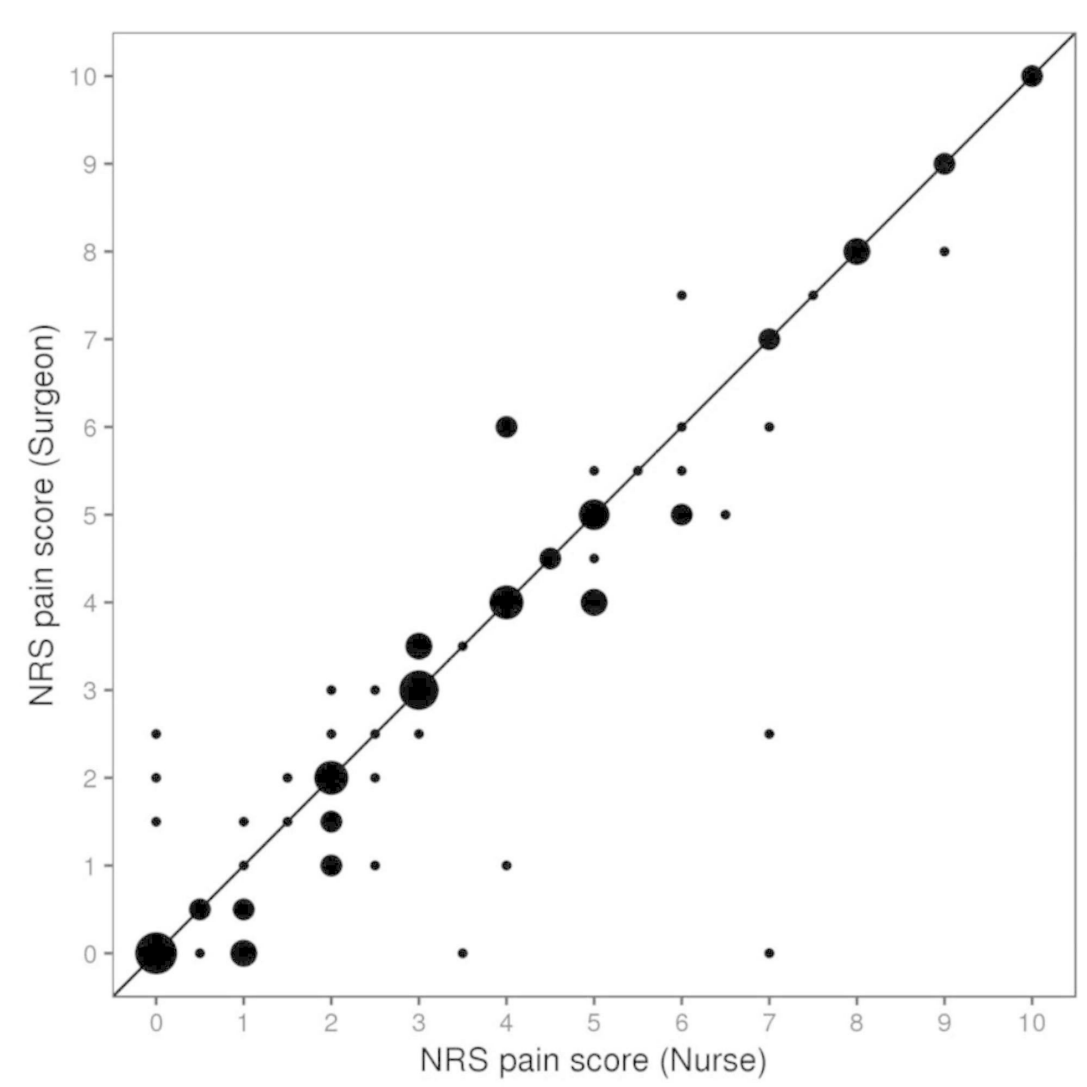
Numerical rating scale (NRS) pain scores reported to the surgeon and nurses The line of equality is shown with a solid 45-degree line. Larger points indicate a higher number of patients at that specific combination of NRS pain scores reported to the surgeon and nurse. The intraclass correlation coefficient equals 0.90 (95% CI: 0.85 - 0.93).

**Figure 2:**
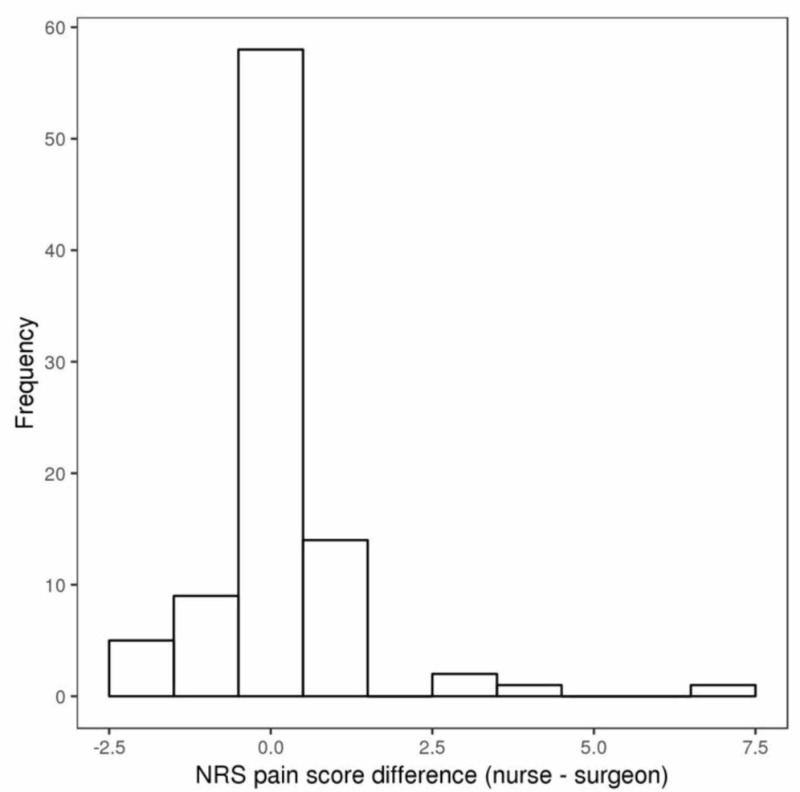
Distribution of differences in numerical rating scale (NRS) pain scores reported to nurses and surgeon (nurse minus surgeon)

Postoperative pain scores reported to surgeons and nurses are summarized in Table 1. The median time between the NRS pain scores that were reported to the surgeon and those that were reported to the nurse was 16 minutes (range: 1 - 59 minutes). The median NRS pain score that was reported to both the surgeon and nursing staff was 3 (range: 0 - 10), and this corresponded to a median difference of 0 (range: -2.5 to 7). Forty-nine patients (54%) reported the same score to the surgeon and nurses, and 79 patients (88%) reported scores to the surgeon and nurses that were within ± 1 point of each other. The estimated ICC of 0.90 (95% CI: 0.85 - 0.93) indicates very good agreement between the reported pain scores (Figures 1-2). The degree of agreement in pain scores reported to surgeons and nurses was fairly similar according to sex and age; the estimated ICC comparing pain scores reported to surgeons and nurses was 0.86 for females, 0.95 for males, 0.93 for patients younger than 65, and 0.86 for patients 65 years of age or older (Table 1).

**Table 1:** Postoperative pain scores reported to surgeons and nurses

Variable	All patients (N=90)	Females (N=50)	Males (N=40)	Age < 65 (N=38)	Age > 65 (N=52)
Time between surgeon and nurse reported pain scores (minutes)	16 (1,59)	17 (1,59)	15 (1, 54)	17 (1, 52)	15 (1, 59)
Postoperative pain score reported to surgeon	3 (0, 10)	4 (0, 10)	2 (0, 10)	4 (0, 10)	3 (0, 10)
Postoperative pain score reported to nurse	3 (0, 10)	4 (0, 10)	2 (0, 10)	4 (0, 10)	3 (0, 10)
Difference between pain score reported to surgeon and nurses	0 (-2.5, 7)	0 (-2.5, 7)	0 (-1.5, 4.5)	0 (-2.5, 4.5)	0 (-2.5, 7)
Equal pain scores reported to surgeon and nurse	49 (54.4%)	26 (52.0%)	23 (57.5%)	24 (63.2%)	25 (48.1%)
Pain scores reported to surgeon and nurse with +/- 1	79 (88.0%)	41 (82.0%)	38 (95.0%)	34 (89.5%)	45 (86.5%)
ICC (95% CI) assessing agreement between pain scores reported to surgeon and nurse	0.90 (0.85, 0.93)	0.86 (0.76, 0.92)	0.95 (0.90, 0.97)	0.93 (0.87, 0.96)	0.86 (0.77, 0.92)
ICC = intraclass correlation coefficient; CI = confidence interval. The sample median (minimum, maximum) is given for continous variables					

**Figure 3:**
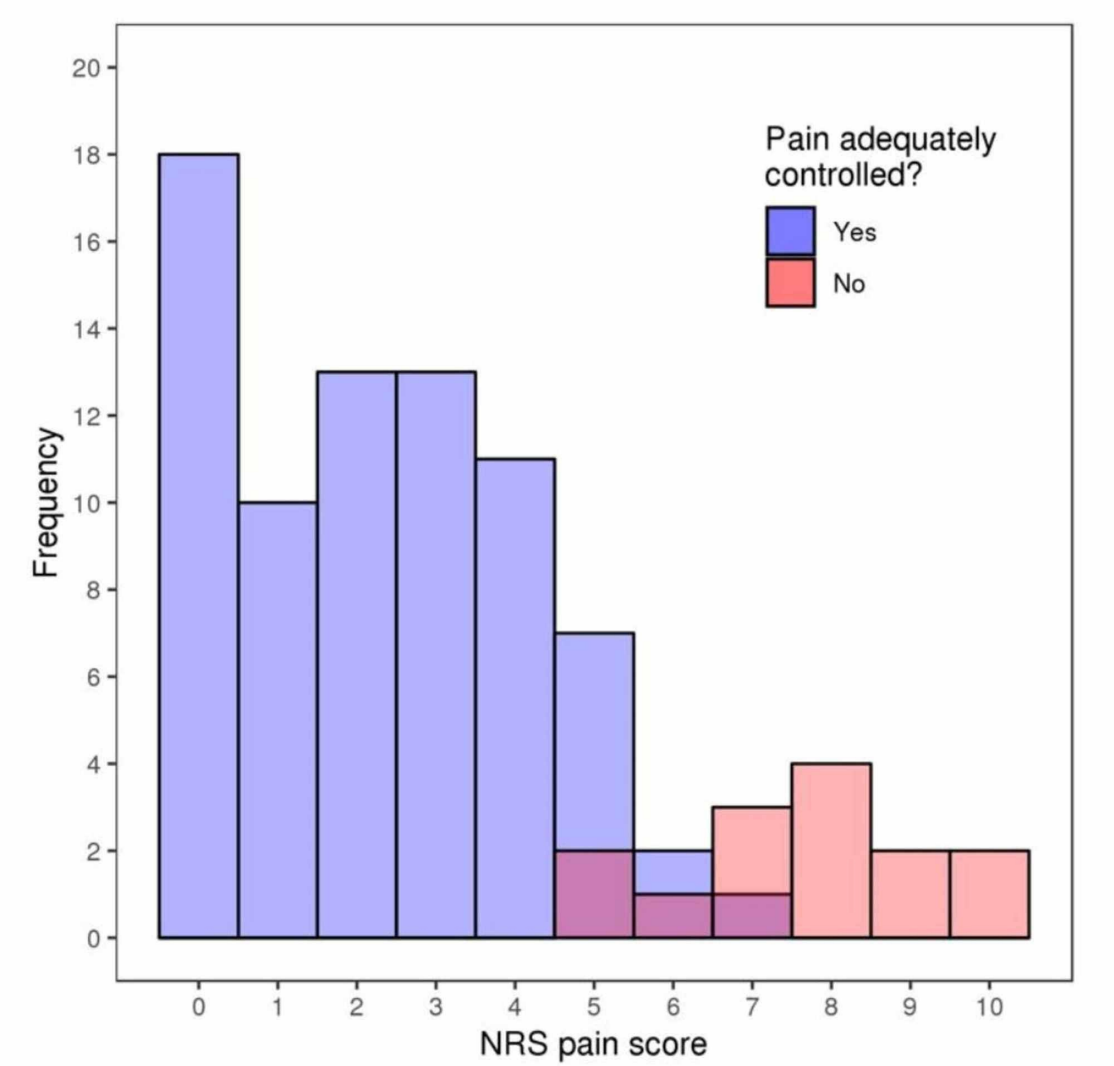
Histogram of the numerical rating scale (NRS) pain scores reported to the surgeon for patients who reported that their pain was controlled (denoted as "Yes") and patients who reported that their pain was not controlled (denoted as "No")

Of the 90 patients, 75 (84.3%) reported that their pain was adequately controlled (this information was not available for one patient). For the secondary aim of the study, we compared the NRS pain score for patients who reported that their pain was or was not adequately controlled. For the patients who reported to the surgeon that their pain was adequately controlled, NRS pain scores reported to the surgeon were significantly lower compared to the pain scores for patients who reported that their pain was not adequately controlled (P< 0.001), with a difference in median NRS pain scores of 5.5 points. It is worth noting, however, that there was some overlap in NRS pain scores for these two patient groups in the 5-7 range (Figure 3).

## Discussion

The reporting of pain relies on subjective scoring systems with no reliable objective measurements available [3-4]. As such, it is unknown if patients report similar scores to both the nursing staff and the physician in the postoperative setting. This becomes important as medical decisions are often based on a patient’s described pain levels. If there are inconsistencies between reporting the pain level to different providers, this may lead to confusion and inadequate care. Additionally, as the medical field begins to tackle the opioid epidemic and as insurance companies continue to explore tying reimbursement to outcomes measures, accurate and consistent reporting of pain control becomes crucial to quality patient care [9-12].

In this study, we prospectively evaluated pain scores reported by the patient to both the surgeon and the nursing staff. This was completed in a blinded fashion in order to reduce bias. We hypothesized that postoperative patients would report similar scores to the nursing staff and physician. Our secondary aim was to compare the pain score and whether the patient felt that their pain was controlled.

Previous research has attempted to compare pain scores reported to the nursing staff compared to the physician during the same clinic visit. Martin et al. retrospectively reviewed initial clinic encounters for 201 patients who were deemed operative candidates. Eighty-one percent of these patients reported a significantly higher score to the physician as compared to the nurse. This difference was dramatic, with an average discrepancy of 2.9 points on the VAS [8]. The same group then reviewed 201 patients who were ultimately treated nonoperatively to see if the difference persisted. They found that the nonoperatively-treated patients continued to report higher pain scores to the physicians as compared to the nursing staff during the same clinic visit, but the degree of difference was less dramatic. Only 53% of patients reported higher pain scores to the physician as opposed to 81% in the first study. The average difference in reported VAS scores was 1 point [7].

Unlike the study by Martin et al., we observed very high agreement between pain scores provided to the nursing staff as opposed to the physician. Specifically, 54% of patients reported the same score to both medical providers and 88% of patients reported a pain score to the nursing staff within 1 point of the score given to the surgeon. A study by Bijur et al. reported the minimum clinically significant difference in pain was 1.3 on the NRS scale, and as previously mentioned almost 90% of the differences observed in our study were under that threshold [13]. In addition, our data demonstrated an interclass correlation coefficient of 0.9, further indicating very good agreement between the reported scores. The interclass coefficient remained high when specifically evaluating patients based on gender or age. Our data, therefore, suggests that physicians may reliably use nursing-collected pain scores to make medical decisions in the postoperative hospital setting.

Our secondary aim evaluated the overall NRS score with the patient’s perception of how well their pain was controlled. We aimed to correlate if the absolute number was a reliable indicator of the patient’s perception of their pain control. We found that there was a significant difference in scores between those who reported that their pain was controlled versus those who reported their pain as uncontrolled. The patients who reported controlled pain had a median 5.5 lower score on the NRS scale compared to those whose pain was uncontrolled. There was, however, overlap in the mid-range of the NRS scale, with some patients reporting a pain level of 5 - 7 as controlled, and some patients reporting this as uncontrolled. This suggests that while the NRS scale is predictive of a patient’s perceived pain levels at the extremes, it becomes less accurate in the mid-range. Currently our practice instructs nurses to give a set amount of medication for patients who report their pain as 3-5 on the NRS scale and an increased dose for patients who report their pain in the 6-10 range. With this current model we may be over-treating patients who report their pain in the 6-7 range and still consider this controlled. Future studies may include adding the patient’s perceived level of pain control to the absolute number in order to determine the appropriate amount of medication to provide to the patient.

Several limitations of this study should be noted. This study was performed at a single institution with patients undergoing mostly elective procedures. It is possible that in a different clinical environment the difference observed between patient-reported pain scores between the members of the medical team would be greater. Additionally, the scores between physician and nursing staff were collected in a relatively short period of time, introducing possible priming of responses to the nursing staff. We attempted to reduce this by allowing enough time (16 minutes on average) for patients to become distracted with other activities while not allowing enough time for pain-inciting events, such as physical therapy, to influence the results.

## Conclusions

In conclusion, the results of this study indicate that there is very good agreement between physician and nursing-obtained verbal numerical rating pain scores, with 88% of the scores at most being 1-point different, demonstrating no clinically significant difference. Additionally, as expected, NRS pain scores were dramatically lower in patients who reported that their pain was adequately controlled in comparison to patients whose pain was uncontrolled. Interestingly, there was some overlap in the reported pain scores between the two groups in the 5-7 range of the NRS scale, suggesting that medical providers should further inquire about the patient’s perceived pain control in this range to avoid over or under-treatment of pain.
